# A Comparative Study of the Application of Photobiomodulation Therapy in the Treatment of Temporomandibular Joint Diseases (TMD): Photobiomodulation Therapy in the Treatment of TMD

**DOI:** 10.31661/gmj.v13iSP1.3644

**Published:** 2024-12-29

**Authors:** Soraya Soleimani, Ehsan Rouhollahpour Ahangar, Mehdi Akafzadeh, Navid Youssefi

**Affiliations:** ^1^ Department of Prosthodontics, Shahid Beheshti University of Medical Sciences, Tehran, Iran

**Keywords:** Photobiomodulation, Temporomandibular Disorder (TMD), Quality of Life

## Abstract

**Background:**

The primary objective of this trial was to evaluate the supplementary effect of photobiomodulation when added to orofacial myofunctional therapy on symptoms of Temporomandibular Disorder (TMD) in TMD.

**Materials and Methods:**

This pilot randomized trial investigated the effects of photobiomodulation on TMD symptoms. Eleven women with mild to moderate TMD were randomly assigned to either an experimental group (EG, n=5) receiving photobiomodulation combined with orofacial myofunctional therapy or a control group (CG, n=6) receiving passive orofacial myofunctional treatment alone. Participants underwent 12 sessions of treatment, with photobiomodulation administered using an 830nm laser at 48J/cm2 fluence. Outcome measures included pain levels and oral health-related quality of life.

**Results:**

The experimental group had considerably greater readings for all motions, including protrusion (P=0.037), sides (P=0.0025; P=0.0014), and opening (P=0.039), according to an examination of the groups. The control group’s findings were statistically significant only for the measurements on the left and right (P=0.0030 and 0.0026, respectively). Numerous traits associated with mandibular mobility showed a discernible improvement before and after therapy in the EG. These variables include noise at the right and left temporomandibular joint (TMJ) during opening and closing of the mouth (P=0.019), noise at the right and left TMJ during protrusion (P=0.147; P=0.049), noise at the right and left TMJ during opening of the mouth (P=0.028; P=0.038), and noise at the left TMJ during opening of the mouth (P=0.012). There was only a reduction in left-sided pain (P=0.019) for the control group when comparing mandibular movements before and after treatment.

**Conclusion:**

In conclusion, photobiomodulation treatment improved orofacial myofunctional therapy results, which increased speech therapy’s efficacy in treating temporomandibular disorders.

## Introduction

Temporomandibular dysfunction (TMD) is a group of conditions that affect the muscles used for chewing, the temporomandibular joint (TMJ), and the functioning of the chewing mechanism [1][2]. Alterations in the temporomandibular joint’s motions are a defining feature of this disorder [3], which can result from a variety of factors, including anatomy, psychology, and harmful habits like nail biting, teeth grinding, and tongue sucking. TMDs are common conditions that can result from various factors, including anatomy, psychology, and habits like nail biting and teeth grinding. TMDs often cause pain, limited jaw movement, joint sounds, muscle soreness, and trouble chewing, significantly impacting quality of life [3][4].

The temporomandibular joint is needed for essential functions like speaking, eating, and swallowing. TMD is a common cause of pain in the face and jaw area. Orofacial myofunctional therapy, a type of speech therapy, is used to treat TMD by improving the function of the stomatognathic system through exercises [5], relaxation, and pain management techniques, aiming to enable comfortable and safe activities like chewing [2][3]. Manipulation, mobilization, and targeted exercises are manual treatment techniques that increase fiber flexibility, promote synovial fluid production, and improve mobility and proprioception [4]. With slow, deliberate manipulations in painful regions, manual therapy also helps release tension, get rid of trigger points, and lessen excruciating feelings associated with dysfunction [5].

The choice of exercise should be carefully evaluated since it may not be appropriate in every situation or stage of the healing process [5]. Improper use might make the sufferer’s agony and suffering worse. The effects of low-level laser therapy on TMD by photobiomodulation have been the subject of several research studies [6][7][8]. The results demonstrate that the technique helps address this issue [6][7][8]. Photobiomodulation has been studied extensively for its capacity to ease pain, promote tissue regeneration, and reduce inflammation in the location where TMJ issues arise [6][7][8].

Low-level laser therapy effectively reduces pain and offers patients quick relief. The laser causes electrons or other molecular components to become active when they enter tissue, which causes charge mobility inside the molecule [8]. Low-level laser therapy stimulates cellular and physiological processes, promoting balance and equilibrium in the body, and can be used to treat various conditions, but requires proper dosage and application by a knowledgeable therapist [7][8][9]. The laser acts as a modifying agent by directly affecting muscle fibers, which lessens discomfort and muscular contraction by encouraging local microcirculation. The patient feels less pain when the trigger point is the focus, which promotes tissue healing and reduces edema and inflammation [1][6][7][8][9]. This non-invasive treatment method can help alleviate this pain and improve the function of the jaw and mouth.

If left untreated, TMDs can lead to decreased quality of life, lost workdays, and increased medical expenses [10][11]. As current treatments for TMD often have limited efficacy and may even exacerbate symptoms if not properly administered, and considering the existing literature on the benefits of photobiomodulation in pain relief and tissue regeneration, we aimed to investigate the supplementary effect of photobiomodulation when added to orofacial myofunctional therapy on symptoms of TMD. This study is novel in that it explores the combined effect of photobiomodulation and orofacial myofunctional therapy, whereas previous studies have primarily focused on the individual effects of these treatments.

## Materials and Methods

### Trial Design

This was a pilot clinical trial study conducted at Department of Prosthodontics of Shahid Beheshti University of Medical Sciences, Tehran, Iran in 2020. Under reference number IET/357089, the Human Research Ethics Committee at the Shahid Beheshti University of Medical Sciences institution accepted this research. On the informed consent form (ICF), volunteers attested to their agreement to participate in the research. The experiment was carried out at a university speech therapy facility.

### Participants

First, a screening was conducted to determine whether volunteers met the study’s eligibility requirements for TMD treatment. The study included female participants with mild to moderate muscular TMD who were not on TMD treatment. The participants were chosen using the research diagnostic criteria for temporomandibular disorders [12]. The research excluded pregnant patients, those undergoing radiation or chemotherapy, those using analgesics or anti-inflammatories continuously for treatment of TMD, and people with moderate to severe class II or III occlusion.

### Interventions

The IBRAMED Laser Pulse Diamond Line apparatus exposed the patients to low-level laser irradiation. This device has a TMJ fluency of 48 J/cm2, a 3J dose, and an 830 nm wavelength. Even though the literature suggested high doses for pain relief and the studies did not specify a dosage, the decision was taken to begin the study with a modest dose to investigate the effects of various dosages in clinical situations [12]. Additionally, the idea was to achieve purposes beyond analgesia, such as enhancing mandibular movements. It’s essential to take safety measures while utilizing low-level laser therapy for photobiomodulation, including wearing protective goggles, shielding your eyes from the laser beam, being aware of reflective surfaces, and maintaining good operating ergonomics. Lasers were employed throughout the sessions, which took place in a private space. The infrared waves were applied to five specific spots on the volunteers’ skin surrounding the TMJ: the masseter, temporalis, sternocleidomastoid, and trapezius muscles; the upper and lower points of the condylar position on the front and back; and the upper and lower points on the side-to-side.

Orofacial myofunctional therapy was administered to the volunteers. The program addressed various subjects, including TMD education, habit breakers, thermotherapy instructions, massage and muscle relaxation techniques for pain relief, mandibular exercises, orofacial function training, and organofunctional exercises for the lips, tongue, and cheeks. Advice, targeted exercises, and personalized functional training were all part of orofacial myofunctional treatments tailored to each patient’s requirements. Proprioception was also emphasized as a way to confront and disrupt negative tendencies. The suggestions after each session included assessing the regularity, kind, and frequency of at-home activities and the consistency of routines.

The orofacial myofunctional therapy protocol consisted of a series of exercises and techniques tailored to each participant’s specific needs, aiming to improve mandibular function, reduce pain and discomfort, and enhance overall orofacial health. The protocol was divided into three phases: Phase 1 (Awareness and Relaxation, Weeks 1-4) focused on exercises such as masseter and temporalis relaxation, mandibular movements, and awareness of the position and movement of the mandible. Phase 2 (Strengthening and Coordination, Weeks 5-8) included isometric exercises to strengthen the muscles of mastication, mandibular coordination exercises, and functional chewing exercises. Phase 3 (Functional Training, Weeks 9-12) emphasized functional chewing, speech exercises, and proprioception exercises to enhance awareness of the position and movement of the mandible. Additional techniques included breathing exercises to reduce stress and promote relaxation, and exercises to coordinate the movement of the mandible with the tongue and lips.

The study was conducted using the participants’s reports on task performance and their presentation to the researcher of how these activities were carried out in their daily routines. At that moment, preparations were also established for the next week. The study groups met for twelve fifty-minute sessions each week. Each session was five minutes for orientation, fifteen minutes for laser treatment, and thirty minutes for orofacial myofunctional therapy.

### Outcomes

Visual analog scale was used to quantify the individuals’ pain levels; it ranges from zero (no pain) to ten (intolerable anguish) [9][13]. To measure how better dental health affected people’s quality of life, researchers employed an updated dental health effect profile using The Oral Health Impact Profile (OHIP-14) questionnaire [14]. Participants completed the OHIP-14 questionnaire, which consists of 14 items assessing the impact of oral health on daily life. Each item is rated on a 5-point Likert scale (0=never, 1=hardly ever, 2=occasionally, 3=fairly often, 4=very often). The questionnaire was divided into subscales of: Functional Limitation, Physical Pain, Psychological Discomfort, Physical Disability, Psychological Disability, and Social Disability.

Mandibular movements were assessed using a standardized protocol. Participants were seated in a comfortable position with their head in a neutral position, and a trained examiner used a digital caliper to record maximum mouth opening, lateral excursion to the right and left, and protrusion. Each measurement was taken three times, and the average value was recorded.

Measurements were performed before interventions and after interventions in 12th week.

### Randomization and Blinding

Using random assignment, the participants were divided into two groups: the experimental group was given even numbers, while the control group was assigned odd numbers. By simulating laser applications utilizing the low-level laser therapy process without providing the light beam, inactive photobiomodulation is achieved. None of the patients in this group received photobiomodulation, even with the device turned on. The volunteers had no idea which group they were supposed to be in.

### Sample Size

Given the pilot nature of this study, a convenience sampling strategy was employed, with all samples collected over a one-year period.

### Statistical Methods

All the gathered information was organized and saved in an electronic spreadsheet using normal data-gathering processes for descriptive statistical analysis, including frequency, central tendency, and inferential analytic measures. For the parametric examination, the student’s t-test for paired samples was used, and the Kolmogorov-Smirnov test was utilized to ascertain whether the data distribution was normal. A significance threshold of 5% was considered appropriate for the statistical difference. All tests were performed in R, version 3.2.2.

## Results

**Figure-1:**
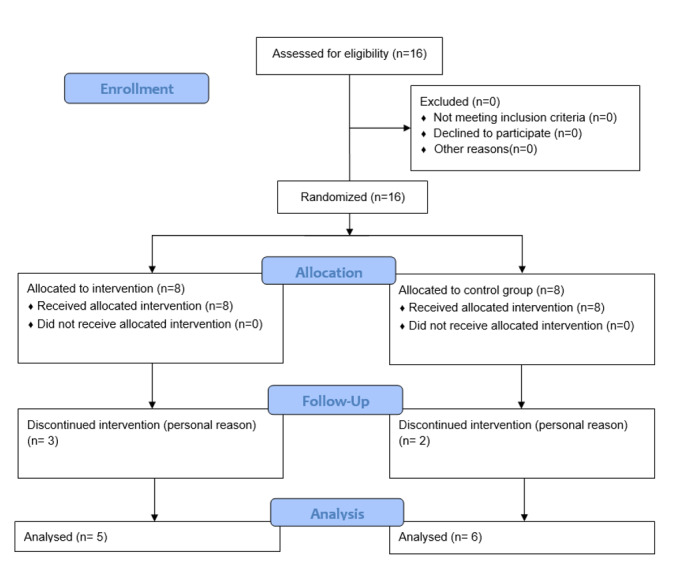
Figure 1. CONSORT flow diagram of studied subjects

**Table:** Table-1. Measurements of Mandibular Movements Before and After Therapy

**Movement**	**Group**	**Pre-therapy**	**Post-therapy**	**P-value**
Opening, mm	Experimental	40.90 ± 8.97	46.98 ± 6.91	0.039
	Control	40.12 ± 5.98	42.98 ± 3.99	0.097
Right side, mm	Experimental	6.98 ± 2.91	10.97 ± 1.39	0.0025
	Control	6.82 ± 2.57	9.56 ± 3.99	0.0026
Left side, mm	Experimental	7.20 ± 2.54	10.56 ± 1.54	0.0014
	Control	7.41 ± 2.31	9.87 ± 1.21	0.003
Protrusion, mm	Experimental	6.87 ± 1.56	9.33 ± 0.75	0.037
	Control	4.98 ± 1.67	6.12 ± 1.32	0.34

Sixteen women were included in the study. During treatment, two volunteers in the control group and three participants in the experimental group discontinued their therapy for personal reasons. The group being investigated consisted of five women who underwent photobiomodulation (EG). As part of the control group, six women had passive orofacial myofunctional treatment, as shown in Figure-1. Eleven participants with ages 25 to 55, with mild to moderately severe TMD affecting both sides of their bodies were finally evaluated. Data on participant pain was analyzed using the Visual Analog Scale for Pain before and after treatments were implemented in the experimental and control groups. Both groups showed a substantial decrease in pain VAS score during the intragroup analysis (EG P=0.002; CG P=0.007). The experimental group’s beginning average was 8.60, and its end average was 1.00. On the other hand, the control group had a final average value of 1.83 after starting at 7.50 on average.

Table-1 compares mandibular movement measures taken before and after treatment for the experimental group (EG) and the control group (CG). The experimental group had considerably greater readings for all motions, including protrusion (P=0.037), sides (P=0.0025; P=0.0014), and opening (P=0.039), according to an examination of the groups. The control group’s findings were statistically significant only for the measurements on the left and right (P=0.0030 and 0.0026, respectively). Numerous traits associated with mandibular mobility showed a discernible improvement before and after therapy in the EG. These variables include noise at the right and left TMJ during opening and closing of the mouth (P=0.019), noise at the right and left TMJ during protrusion (P=0.147; P=0.049), noise at the right and left TMJ during opening of the mouth (P=0.028; P=0.038), and noise at the left TMJ during opening of the mouth (P=0.012). There was only a reduction in left-sided pain (P=0.019) for the control group when comparing mandibular movements before and after treatment. Table-2 compares the participants’ quality of life scores before and after treatments between the experimental group (EG) and the control group (CG). The seven assessment aspects where the experimental group showed significant improvement were functional limitation (P=0.044), physical pain (P=0.005), psychological discomfort (P=0.005), physical limitation (P=0.0021), psychological limitation (P=0.033), social limitation (P=0.011), and disability (P=0.025). Overall, the experimental group’s quality of life significantly improved, and the OHIP-14 protocol’s total score showed an especially significant improvement (P=0.0002). The CG’s overall quality of life significantly improved after the surgery (pP=0.013). Improvements were seen in physical restriction (P=0.039), psychological discomfort (P=0.002), and physical pain (P=0.00001).

The change in OHP scores (Δ) is significantly different between the two groups. The Experimental group showed a greater reduction in OHP scores (-21.66) compared to the Control group (-16.01), with a P-value of 0.04.

## Discussion

**Table:** Table2. Measurement of Quality of Life (OHIP-14) Before and After Treatment

**Parameter**	**Group**	**Pre-therapy**	**Post-therapy**	**P-value**
**Functional Limitation**	Experimental	2.39 ± 1.23	0.78 ± 0.43	0.044
	Control	1.47 ± 1.54	0.00 ± 0.00	0.087
**Physical Pain**	Experimental	5.32 ± 2.16	0.77 ± 0.23	0.005
	Control	6.01 ± 1.08	1.23 ± 1.11	0.00001
**Psychological Discomfort **	Experimental	5.99 ± 1.99	1.67 ± 1.29	0.005
	Control	5.44 ± 2.14	2.13 ± 1.43	0.002
**Physical Limitation**	Experimental	5.11 ± 1.26	0.59 ± 0.77	0.0021
	Control	3.49 ± 2.13	1.12 ± 1.43	0.039
**Psychological Limitations **	Experimental	3.44 ± 1.99	0.86 ± 0.76	0.033
	Control	2.78 ± 2.45	1.76 ± 1.77	0.341
**Social Limitations**	Experimental	2.11 ± 1.13	0.86 ± 1.22	0.011
	Control	1.67 ± 2.79	0.21 ± 0.29	0.34
**Disability**	Experimental	1.32 ± 0.77	0.00 ± 0.00	0.025
	Control	1.67 ± 2.65	0.00 ± 0.00	0.187
**Total OHIP**	Experimental	26.98 ± 6.78	5.32 ± 4.41	0.0002
	Control	22.99 ± 12.98	6.98 ± 4.76	0.0013
**Δ (After - Pre)**	Experimental	-	-16.01 ± 7.43	0.004
	Control	-	-21.66 ± 5.31

The results showed that both the experimental group (EG) and control group (CG) experienced significant reductions in pain, with the EG showing a more substantial decrease. The EG also demonstrated significant improvements in mandibular movement measures, including protrusion, opening, and lateral movements, as well as a reduction in TMJ noise. Additionally, the EG showed significant improvements in quality of life, with improvements in functional limitation, physical pain, psychological discomfort, physical limitation, psychological limitation, social limitation, and disability. The control group also showed some improvements, but to a lesser extent. Notably, the EG showed a greater reduction in the total Oral Health Impact Profile (OHIP) score, with a decrease of 21.66 points (from 26.98 to 5.32), compared to a decrease of 16.

Similarly, a study by Dias et al. (2022) found that photobiomodulation combined with orofacial myofunctional therapy improved the quality of life of individuals with TMD [15]. Another study by Alves et al. (2021) found that photobiomodulation associated with orofacial myofunctional therapy improved temporomandibular joint dysfunction [16]. However, our study had a smaller sample size compared to the study by Dias et al. (2022), which had 34 volunteers. Additionally, our study only investigated the effects of PBM on pain levels and oral health-related quality of life, whereas the study by Alves et al. (2021) investigated the effects of photobiomodulation on temporomandibular joint dysfunction. In contrast, a systematic review by Altuhafy et al. (2024) found that the evidence for the effectiveness of photobiomodulation combined with orofacial myofunctional therapy in orofacial pain disorders is limited, and further randomized controlled trials with extended follow-up periods are needed to obtain firm conclusions [17].

Our study found that the experimental group had considerably greater readings for all motions, including protrusion, sides, and opening and significantly lower pain scores. Its similar to findings of a systematic review and meta-analysis by Hanna et al. (2021) that found that photobiomodulation significantly reduced pain intensity, improved maximum mouth opening (MMO), and increased pressure pain threshold (PPT) in patients with TMD [18]. Similarly, a systematic review by Farshidfar et al. (2022) reported that photobiomodulation alleviated pain and improved MMO in patients with TMD [19]. However, the studies varied in their methodological quality. Therefore, further high-quality studies are needed to confirm the efficacy of photobiomodulation in treating TMD. Nonetheless, the available evidence suggests that PBMT is a safe and effective treatment option for TMD, and its use in combination with other therapies may enhance its benefits.

## Conclusion

The conclusions of this pilot study demonstrated that the combination of laser therapy and orofacial myofunctional therapy improved the treatment of temporomandibular muscle problems statistically.

## Conflict of Interest

There are no conflicts of interest.
